# Enfortumab vedotin in metastatic urothelial carcinoma: the solution EVentually?

**DOI:** 10.3389/fonc.2023.1254906

**Published:** 2023-09-13

**Authors:** Brigida Anna Maiorano, Martina Catalano, Evaristo Maiello, Giandomenico Roviello

**Affiliations:** ^1^ Oncology Unit, IRCCS Casa Sollievo della Sofferenza, San Giovanni Rotondo, Italy; ^2^ Department of Health Sciences, Section of Clinical Pharmacology and Oncology, University of Florence, Florence, Italy

**Keywords:** enfortumab vedotin, ADC, antibody-drug conjugate, nectin-4, urothelial carcinoma, bladder cancer

## Abstract

Metastatic urothelial carcinoma (mUC) is an aggressive malignancy with a dismal prognosis. Enfortumab vedotin (EV) is an antibody-drug conjugate consisting of an antibody targeting Nectin-4. This protein is highly expressed in UC cells. After binding, monomethyl auristatin E is released into cells, causing UC cell death. EV has been approved as a single agent for pre-treated mUC, with interesting improvements in response rate and survival in a setting with limited treatment options. More recently, EV approval occurred in cisplatin-ineligible naïve mUC patients in combination with pembrolizumab. Our review aims to summarize the pharmacological properties, clinical studies, and future developments of EV in mUC.

## Introduction

Urothelial carcinoma (UC) is the tenth most common cancer worldwide. Only 5% of cases are diagnosed in the metastatic setting. However, around 50% of patients who underwent cystectomy for a localized disease will eventually develop metastases (1). In the metastatic setting, the sequence of platinum-based chemotherapy (CT) followed by maintenance avelumab represents the standard of care nowadays, after improving overall survival (OS) and progression-free survival (PFS) compared to platinum-based CT plus best supportive care (2). Immune checkpoint inhibitors (ICIs) are also used in the platinum-progressive setting, improving survival over the alternative CT regimens, mainly taxanes and, in Europe, vinflunine (3–7). Moreover, ICIs - pembrolizumab and atezolizumab - are approved in the first line for platinum-ineligible patients (8–10). However, despite considerable progress, metastatic UC (mUC) remains incurable, as over half of patients do not respond to treatments. Intrinsic or acquired resistance to CT and ICIs leads to a median OS (mOS) that still now does not overcome 21 months, with a 5yr survival rate <10% (1, 11). Less than 1 out of 5 mUC patients carries mutations of the Fibroblast-growth factor receptors (FGFR) and is a candidate for FGFR inhibitors, such as erdafitinib, in the platinum-progressive setting (12–14). However, no effective treatments besides these regimens have been recently approved. In this scenario, antibody-drug conjugates (ADCs), such as enfortumab vedotin (EV) and Sacituzumab govitecan (SG), have been studied to fill the gap of survival and response of mUC patients. ADCs are small molecules with an anticancer drug linked to a monoclonal antibody: the antibody binds the cell surface receptors, and then the anticancer drug is released into the intracellular environment (15).

Our review focuses explicitly on the EV, which has been approved by the Food and Drug Administration (FDA) and the European Medical Agency (EMA) for mUC patients progressing to platinum and ICIs (16, 17). We describe the pharmacological characteristics and the clinical studies of EV in mUC, outlining its future developments.

## Pharmacology of EV

Nectin-4, also called Poliovirus receptor-like 4 (PVRL4), is a type I transmembrane protein acting as an immunoglobulin-like cell adhesion molecule (18). While normal bladder tissue displays a weak-to-moderate expression of Nectin-4, this is highly expressed by UC cells, as it contributes to the growth and spread via the activation of WNT-beta-catenin and Rac proteins of the Phosphoinositide 3-kinases/Protein kinase B (PI3K-AKT) pathway. Due to the higher expression in UC tissue, Nectin-4 represents a promising target for drug design (19).

**Figure 1:**
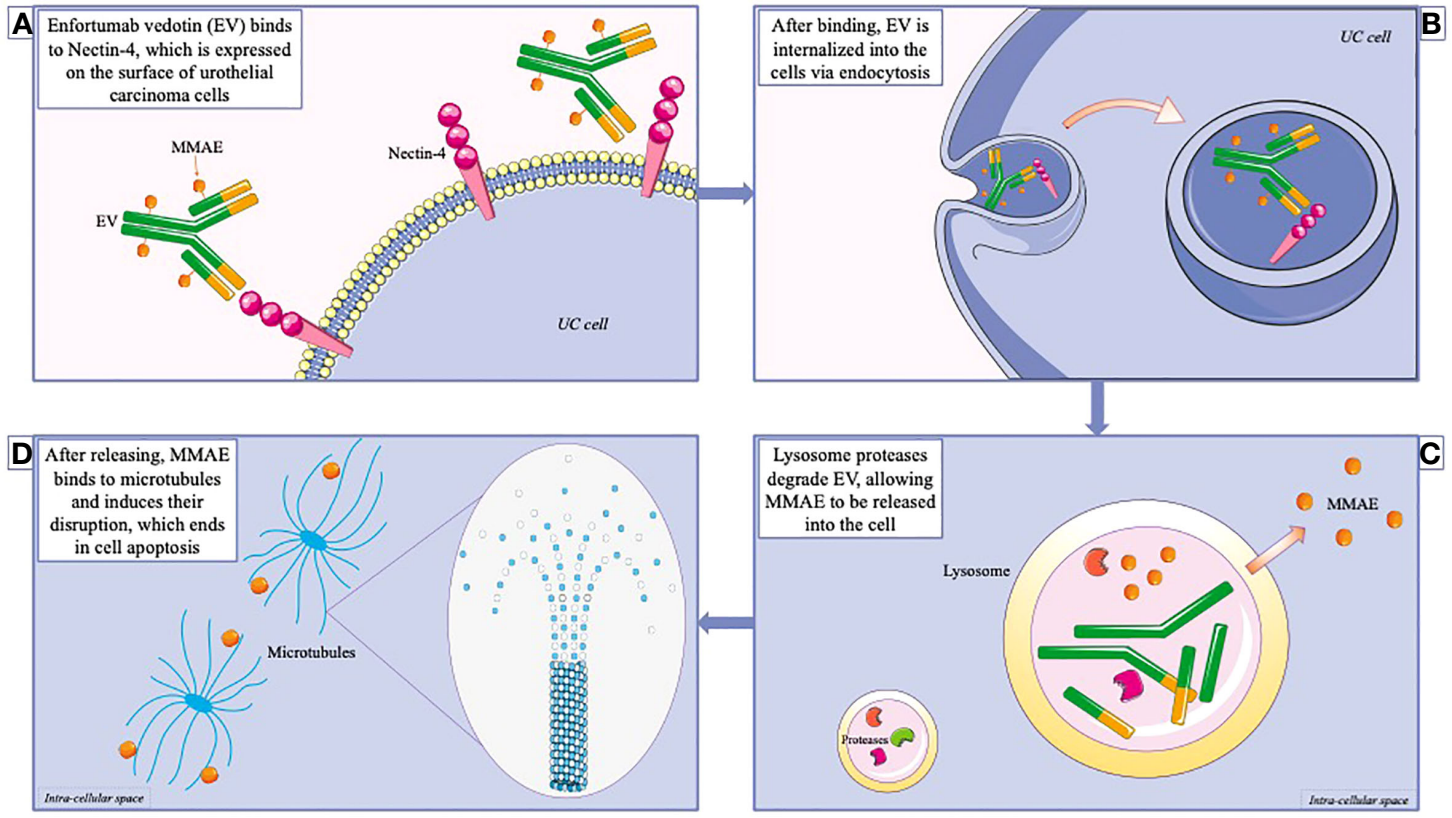
Mechanism of action of enfortumab vedotin (EV). **(A)** EV is an antibody-drug conjugate, consisting in an antibody conjugate with the cytotoxic agent Monomethyl auristatin E (MMAE). EV binds to nectin-4, which is highly expressed on urothelial cancer cells. **(B)** The complex is subsequently internalized into the cells via endocytosis. **(C)** After reaching the lysosomes, EV is degraded, allowing MMAE to be released into the intracellular district. **(D)** MMAE acts as a microtubules disruptor, therefore interfering with mitosis, and leading to cell apoptosis.

EV is an ADC containing a fully human IgG1 monoclonal antibody targeting Nectin-4, plus a protease-cleavable payload, monomethyl auristatin E (MMAE). The Adc is internalized after binding the extracellular domain of Nectin-4 with high affinity. MMAE is broken down by proteases and released into the cells, disrupting microtubules, blocking mitosis, and finally inducing apoptosis (**Figure 1**).

EV has a half-life of around 3.4 days and is metabolized by the Cytochrome P450 3A4 (CYP3A4) liver enzyme. The excretion of MMAE is in feces (17%) and urine (6%). An increase of area under the curve (AUC) of around 50% has been evidenced in case of hepatic impairment, but no modifications have been observed in case of renal impairment (16, 17).

## Clinical studies of EV in mUC

Several trials have evaluated EV in various UC settings in recent years, leading to its approval in metastatic disease (**Table 1**).

**Table 1:** Clinical trials results of enfortumab vedotin in metastatic urothelial cancer.

Study (ref)	Phase	No of pts	Treatment	Patients	Setting	Tumor site	ORR (%)	CR (%)	PFS (mo.)	OS (mo.)	AEs ≥G3
**EV-101** ^20^	I	112	EV (dose escalation up to 1.25 mg/kg) on days 1, 8, 15 of 28-days cycle	Nectin-4 positive metastatic solid tumors or mUC	After ≥ 1 line of therapy	Bladder 71% UTUC 24.5% Others 4.5%	43	5	5.4	12.3	34%
**Japan study** ^22^	I	17	EV 1.0 mg/kg (arm A) or 1.25 mg/kg (arm B) on days 1,8,15 of 28-days cycle	Japanese patients with mUC	After platinum regimen or platinum ineligible	Bladder 70.6% UTUC 19.6%	44.4 (A) 25 (B) 35.3 (A+B)	1 (A) 0 (B)	–	–	17.64
**EV-201** ^23^	II	125	EV 1.25 mg/kg on days 1, 8, 15 of 28-day cycle	mUC	After prior platinum and ICI	Bladder 65% UTUC 35%	44	12	5.8	11.7	54
**EV-201** ^24^	II	89	EV 1.25 mg/kg on days 1, 8, 15 of 28-day cycle	mUC (platinum ineligible)	After prior ICI	Bladder 57% UTUC 43%	52	20	5.8	14.7	55
**EV-301** ^25^	III	608 (301 EV vs. 307 CT)	EV 1.25 mg/kg on days 1, 8, 15 of 28-day cycle vs CT (docetaxel or vinfluvine or paclitaxel) on day1 of 21-day cycle	mUC	After prior platinum CT and ICI	EV: Bladder 67.4% UTUC 32.6% CT: Bladder 65.1% UTUC 34.9%	40.6 vs. 17.9	4.9 vs. 2.7	5.55 vs. 3.71	12.88 vs. 8.97	51.4 vs. 49.8
**EV-103** ^27^	Ib/II	45	EV 1.25 mg/kg days 1 and 8 and pembrolizumab 200 mg day 1 of 21-days cycle	mUC cisplatin ineligible	First line	Bladder 66% UTUC 33%	73.3	17.8	12.3	26.1	58

AEs, adverse events; no, number; CR, complete response; CT, chemotherapy; EV, enfortumab vedotin; mo, months; mUC, metastatic urothelial cancer; ORR, overall response rate; PFS, progression free survival; pts: patients; OS, overall survival; UTUC, upper urinary tratc urothelial cell carcinoma.

### Platinum- and ICIs- progressing setting

The phase I EV-101 study demonstrated the clinical activity of EV in pre-treated Nectin-4 positive mUC patients. After the dose-escalation phase, a dose-expansion study was performed with the dosage of 1.25 mg/kg on days 1, 8, and 15 of a 28-day cycle. 155 mUC patients were included; 71% had bladder cancer, and the others had upper-tract UC (UTUC). Safety, tolerability, and pharmacokinetic profile were assessed as primary objectives. The overall response rate (ORR) was 43%, with 5% complete responses (CRs). The median PFS (mPFS) was 5.4 months, and the mOS 12.3 months. 51.8% of patients were alive after one year, with a median duration of response (mDoR) of 7.4 months. The most common adverse events (AEs) were fatigue (53%), alopecia (46%), decreased appetite (42%), dysgeusia, nausea, peripheral sensory neuropathy (38% each), pruritus (35%), diarrhea (33%), and maculopapular rash (27%). G3 or higher (≥G3) AEs occurred in 34% of patients, with hyperglycemia in >5%. The discontinuation rate due to AEs was 10%, most commonly due to peripheral sensory neuropathy (20). Based on these results, in 2018, the FDA granted the breakthrough designation for EV in mUC after ICIs progression (21). In the NCT03070990 phase I study conducted in the Japanese population, an ORR of 35.3% and a disease control rate (DCR) of 76.5% were recorded. In this population, dysgeusia and alopecia were the most common AEs (52.9% each), followed by pruritus and dry skin (47.1%), anemia and decreased appetite (41.2%), and fever (35.3%) (22).

Subsequently, the phase II EV-201 (NCT03219333) was designed, and divided into Cohort 1 - including patients pre-treated with both platinum-based CT and ICIs, and Cohort 2 - which included patients pre-treated with only ICIs (23, 24). In Cohort 1, 125 patients were included. Most were heavily pre-treated, with a median number of 3 prior therapies. After a median follow-up (mFU) of 10.2 months, ORR (the primary endpoint) was 44%, with 12% CRs. The mDoR was 7.6 months. Responses were independent of programmed death-ligand 1 (PD-L1) status, previous response to ICIs, number of previous lines of treatment, and sites of metastases. No differences were recorded between high and low expression of Nectin-4. Most responses occurred rapidly (within the first three months). Median PFS was 5.8 months, mOS 11.7 months. Fatigue (50%), alopecia (49%), peripheral neuropathy (40%), decreased appetite or dysgeusia (40%), and rash (22%) were the most common AEs. 44% of patients experienced ≥G3 AEs, among which the most common were neutropenia (8%), anemia (7%), fatigue (6%), and rash (4%). Peripheral neuropathy was the most frequent cause of dose reduction (9%) or discontinuation (6%). 3 out of 4 patients recovered from skin rash and peripheral neuropathy (23).

In the EV-301 phase III trial, patients who progressed to previous platinum-based CT and an ICI were randomized to EV (1.25 mg/kg, days 1, 8, 15 q28; n=301) versus CT (n=307) at the investigator’s choice between docetaxel, paclitaxel, and vinflunine. The study met its primary endpoint, as EV prolonged OS compared with CT (mOS 12.88 vs. 8.97 months; hazard ratio [HR] 0.70, 95% confidence interval [CI], 0.56-0.89; p=0.00142). EV also prolonged mPFS to 5.55 vs. 3.71 months (HR 0.62, 95% CI, 0.51-0.75, p<0.00001). Of note, 98 patients that received EV, and 107 CT, had UTUC: in these patients, OS results were not statistically significant (HR 0.85, 95% CI, 0.57-1.27). The benefit of EV was consistent among all the subgroups, including patients with liver metastases, heavily pre-treated, >65 and >75 years old. ORR was 40.6% vs. 17.9%, and CRs were 4.9% vs. 2.7%. The AEs rate was similar between EV (93.9%) and CT (91.8%). The most common AEs with EV were alopecia (45.3%), peripheral sensory neuropathy (33.8%), pruritus (32.1%), fatigue (31.1%), decreased appetite (30.7%), diarrhea (24.3%), dysgeusia (24.3%), and nausea (22.6%). Hematological toxicity was better than chemotherapy (anemia 11.5%, neutropenia 6.8%). Maculopapular rash (7.4%) and fatigue (6.4%) were the most common ≥G3 AEs. Skin rash (7.4%), peripheral neuropathy (3%), and hyperglycemia were AEs of interest for EV. A skin rash occurred in 43.9% of patients treated with EV. There were seven deaths in the EV group and three in the CT group (25). The survival benefit of EV in this study led to its early stopping and therapy approval.

### ICI-progressing setting

In Cohort 2 of EV-201, 89 ICI-progressing platinum-ineligible patients received EV. After a median follow-up of 13.4 months, ORR (the primary endpoint) was 52%, with a CR rate of 20%. The efficacy was confirmed in all subgroups. mDOR was 10.9 months, mPFS 5.8 months, and mOS 14.7 months. ≥G3 AEs occurred in 55% of patients, with neutropenia (9%), maculopapular rash (8%), and fatigue (7%) as the most common. 46% of patients required a dose reduction and 16% discontinuation. Three deaths (3%) occurred after EV (24). EV filled an important gap in this setting, as it was effective as second-line treatment in platinum-ineligible patients, and representing a valid treatment option in cases with limited strategies. Even without a direct comparison due to the study design, EV emerged as a treatment with a high response rate in nearly half of mUC patients.

### First-line setting

In pre-clinical models, ADCs amplify the ICIs effect, as they trigger immunogenic cell death, making combining the two drugs potentially more effective than the single agent treatment (26). In the phase Ib/II EV-103 study (NCT03288545), EV plus pembrolizumab and/or CT was administered. The study had safety as the primary endpoint; recommended dose, ORR, DCR, DoR, PFS, OS, pharmacokinetics, and biomarkers were secondary endpoints. In cohort A, 45 cisplatin-ineligible naïve mUC patients were treated with EV (1.25 mg/kg, days 1, 8 q3w) plus pembrolizumab (200 mg q3w). After a median follow-up of 11.5 months, ORR was 73.3%, and 17.8% of patients experienced a CR. DCR was 93.3%, with 53.7% of responses lasting >12 months. 81.6% of patients were alive at one year; the 24-mos OS rate was 56.3%, the mDoR was 25.6 months, and the mPFS 12.3 months. The most common AEs were fatigue (51%), alopecia (49%), and neuropathy (56%). 58% of cases developed ≥G3 AEs, mainly lipase increasing (18%), rash (13%), hyperglycemia (7%), and peripheral neuropathy (4%). 13% of patients discontinued the treatment, most commonly due to peripheral sensory neuropathy (27). Based on these data, in 2020, the FDA designated the combination of EV and pembrolizumab as a breakthrough therapy for cisplatin-ineligible untreated mUC patients (28). The EV-302 phase III trial (NCT04223856), evaluating the combination of EV with pembrolizumab versus platinum plus gemcitabine in untreated mUC patients, is ongoing.

### EV safety management and dose modifications

The recommended dose of EV is 1.25 mg/kg (up to 125 mg for patients with a body weight >100 kg) as a single agent on days 1, 8, 15 of a 28-days cycle until disease progression or unacceptable toxicity. In combination with pembrolizumab (200 mg q3w), the recommended dose of EV is 1.25 mg/kg (maximum 125 mg) on days 1. 8 of a 28-days cycle. Three dose reductions have been scheduled, 1.0 mg/kg (maximum 100 mg), 0.75 mg/kg (maximum 75 mg), and 0.5 mg/kg (maximum 50 mg).

Although EV is generally safe and most AEs are mild-to-moderate, patients must be closely monitored due to non-common AEs experienced in the mUC daily practice, such as hyperglycemia, an off-target effect whose etiology remains unclear, leading to diabetic ketoacidosis in patients with and without pre-existing diabetes mellitus. Blood glucose levels should be closely monitored, and EV should be withheld in case of levels >250 mg/dL (17, 23, 29).

Skin rash is a typical AE from EV; its occurrence is mainly due to the cutaneous expression of Nectin-4. The most common manifestations of skin reactions are maculopapular rash and pruritus; however, there is the potential for atypical but severe skin AEs, such as Steven-Johnson syndrome (SJS) and toxic epidermal necrosis (TEN). In the EV clinical trials, the median time to onset of cutaneous AEs was within the first cycle. There is a warning to immediately withhold EV in case of suspected SJS/TEN, and permanently discontinue the drug if the diagnosis is confirmed. Patients should be closely monitored for skin reactions. Topical corticosteroids and antihistamines can be considered for less severe skin manifestations (17, 23, 29).

Peripheral neuropathy can be attributed to microtubule disruption at the interphase. Peripheral neurotoxicity presented around the fifth month of therapy. 3 out of 4 patients had a complete resolution of symptoms. EV should be withheld in case of this AE appearance, until the recovery to G1 and interrupted if >G3 (17, 29, 30).

Other AEs presenting with EV were loss of appetite and interstitial lung disease (which more frequently presented during the first 6 months of treatment). Ocular disorders most commonly involve the cornea, with dry eye symptoms such as keratitis, blurred vision, increased lacrimation, conjunctivitis, limbal stem cell deficiency, and keratopathy. Artificial tears could be used for prophylaxis, and ophthalmic topical steroids can be administered (17).

## Discussion

Still, nowadays, mUC remains an incurable disease. However, in the last decade, a wide approval of different drugs with innovative mechanisms of action has occurred, which has leaped forward survival. EV has demonstrated an impressive efficacy, with one out of two patients responding to the treatment and another one out of four-remaining stable, and having a large group of suitable patients. The efficacy is interesting in patients with visceral metastases, notably in liver metastases representing a poor prognostic phenotype. Using agents targeting a very common ligand, such as Nectin-4, seems an effective strategy to treat mUC, as the tumor tissue highly expresses this target. However, the presence of Nectin-4 on the cell surface alone does not guarantee a response; thus, there is no role of Nectin-4 as a predictive biomarker (31). Given the efficacy results of EV in mUC, this agent has deserved a double breakthrough designation by the FDA as a single agent in pre-treated patients and in combination with pembrolizumab for naïve cisplatin-ineligible cases (16, 21). Results of further combinations are expected. EV represents a valid option that – together with novel agents, such as FGFR inhibitors, ICIs, and other ADC, is expanding the treatment options for mUC patients.

Quality-of-life (QoL) data from the EV-301 trial did not demonstrate a lower global health status with EV than CT. On the contrary, patients had a more significant reduction in pain with EV than with CT (32). Notably, EV is safe in patients with renal impairment, a common comorbidity in mUC. This observation paves the way for the potential use of EV in the neoadjuvant setting, given the need for more effective treatments in localized disease, even for cisplatin-ineligible patients (17).

Different receptors are under investigation as potential ADC targets in mUC, and studies demonstrate no cross-resistance between these pathways. The other ADC, SG, which is directed against TROP2 and has an active metabolite of irinotecan and topoisomerase I inhibitor, has been evaluated in mUC (33). Data from the TROPHY-U-01 study have demonstrated acceptable response rates and survival improvement in different cohorts. Interestingly, the downregulation of Nectin-4 does not co-occur with TROP2, also after EV (34). Based on this evidence, a phase I study (NCT04724018) combines EV and SG for potential synergism. Human epidermal growth factor receptor (HER)-2 is another potential target of ADC in mUC. In a phase II study, the HER-2-targeted ADC, disitamab vedotin, demonstrated a response in half of mUC patients (35).

The approval of EV calls forth the question of the ideal treatment sequencing in mUC. For this purpose, predictive biomarkers and resistance mechanisms should be elucidated. For example, mutations of receptors and transporters of ADC could reduce the payload efficacy and internalization of ADC into the cells. Similarly, the downregulation of transporters after continuous exposure to ADCs could reduce the available targets for the drugs. Finally, tumor microenvironment cells could contribute to the resistance to ADC due to the capability of altering the afflux of inflammatory, angiogenic, and immune cells (36).

Therefore, combining ADCs with agents having different action mechanisms could help overcome potential resistance strategies. EV encouraging response and manageable toxicity profile support its use in various settings, from the neoadjuvant to heavily pretreated patients and in several combinations. The immunogenic cell death induced by EV led to the evaluation of the EV and ICIs combination treatment. Exciting results come from the combination of EV and pembrolizumab that could change the first-line standard of care in the following years (27). In the late-line setting, several early-phase studies test novel combinations with EV, including erdafitinib, SG, cabozantinib, gemcitabine, and saracatinib (**Table 2**).

**Table 2:** Ongoing clinical trials of enfortumab vedotin in metastatic urothelial cancer.

Study	Status	Phase	Estimated enrollment	Treatment	Setting	Primary endpoints
**NCT 04223856**	Recruiting	III	990	EV+pembro vs. CT alone	Previously untreated locally advanced or mUC	PFS OS
**NCT 03288545**	Active, not recruiting	I/II	457 (actual enrollment 348)	EV+pembro in cis ineligible 1L (cohort A) EV+cis in 1L (cohort D) EV+carbo in 1L (cohort E) EV+gem in 1L and 2L (optional cohort F) EV+platinum+pembro in 1L (cohort G) EV+pembro (randomized cohort K)	Locally advanced or mUC	AEs ORR pCR
**NCT 04963153**	Recruiting	I	30	EV+erdafitinib	After platinum and ICI for mUC with FGFR2/3 genetic alterations	AEs Recommended phase II dose MTD of EV
**NCT 05524545**	Recruiting	I	30	Evorpacept+EV and/or other anticancer therapies	After platinum and ICI	DLTs AEs
**NCT 04878029**	Recruiting	I	32	Cabozantinib+EV	After platinum (if eligible) and ICI	AEs
**NCT 04724018**	Recruiting	I	24	SG+EV	After platinum and ICI	MTD of SG plus EV DLT in mg of SG plus EV
**NCT 03288545**	Active, not recruiting	I/II	457 (actual enrollment 348)	EV+pembro in 2L (cohort B) EV+gem in 1L and 2L (optional cohort F)	Locally advanced or mUC	AEs ORR pCR
**NCT 03606174**	Completed	II	425 (actual enrollment 260)	EV+sitravatinib+pembrolizumab	After ICI and platinum	ORR
**NCT 03869190**	Recruiting	Ib/II	645	Atezolizumab+EV	Second line	ORR pCR
**NCT04995419**	Active, not recruiting	II	40	EV	After platinum CT and ICI	ORR PK of EV

AEs, adverse events; carbo, carboplatin; cis, cisplatin; CT, chemotherapy; DLT, dose limiting toxicity; EV, enfortumab vedotin; FGFR, fibroblast growth factor receptor; ICI, immune checkpoint inhibitors; MTD, maximum tolerate dose; mUC, metastatic urothelial cancer; ORR, overall response rate; OS, overall survival; pembro, pembrolizumab; pCR, patological complete response; PFS, progression free survival; PK, pharmacocinetik; SG, sacituzumab govitecan; 1L, first line; 2L, second line.

## Conclusions

EV is an effective and tolerable ADC with exceptional activity in treating mUC patients. Although serious skin rash is a rare but potentially severe side effect that may occur early, close monitoring during the initial cycles is recommended. Peripheral neuropathy is a common side effect that can limit the dose in patients responding well to treatment. In contrast to platinum-based CT, EV appears to be less nephrotoxic and well tolerated in patients with impaired renal function, particularly relevant for those with UTUC. The mechanisms of response and resistance to EV remain a significant area of research with a particular interest in biomarker identification. EV plus pembrolizumab is a promising combination regimen that is well-tolerated and effective in cisplatin-ineligible patients. Although the optimal combination and sequencing of EV with other agents (e.g., CT, targeted therapy, ICIs, and ADCs) are still unknown, EV will be able to expand the options for treating patients with mUC in the next few years.

## Author contributions

BM: Conceptualization, Data curation, Formal Analysis, Investigation, Methodology, Project administration, Software, Validation, Visualization, Writing – original draft, Writing – review & editing. MC: Data curation, Validation, Visualization, Writing – original draft. EM: Funding acquisition, Validation, Visualization, Writing – review & editing. GR: Supervision, Validation, Visualization, Writing – original draft.
